# GRP94 Is Involved in the Lipid Phenotype of Brain Metastatic Cells

**DOI:** 10.3390/ijms20163883

**Published:** 2019-08-09

**Authors:** Naiara Santana-Codina, Anna Marcé-Grau, Laia Muixí, Claudia Nieva, Mónica Marro, David Sebastián, Juan Pablo Muñoz, Antonio Zorzano, Angels Sierra

**Affiliations:** 1 Biological Clues of the Invasive and Metastatic Phenotype Group, Bellvitge Biomedical Research Institute (IDIBELL), L’Hospitalet de Llobregat, E-08908 Barcelona, Spain; 2 Universitat Autònoma de Barcelona (UAB), Campus Bellaterra, Cerdanyola del Vallés, E-08193 Barcelona, Spain; 3 ICFO-Institut de Ciències Fotòniques, The Barcelona Institute of Science and Technology, Carl Friedrich Gauss 3, 08036 Barcelona, Spain; 4 Institute for Research in Biomedicine (IRB Barcelona), The Barcelona Institute of Science and Technology, 08036 Barcelona, Spain; 5 Departament de Bioquímica i Biomedicina Molecular, Facultat de Biologia, Universitat de Barcelona, 08028 Barcelona, Spain; 6 CIBER de Diabetes y Enfermedades Metabólicas Asociadas (CIBERDEM), Instituto de Salud Carlos III, 08028 Barcelona, Spain; 7 Laboratory of Molecular and Translational Oncology, Centre de Recerca Biomèdica CELLEX-CRBC-Institut d’Investigacions Biomèdiques August Pi i Sunyer-IDIBAPS, E-08036 Barcelona, Spain

**Keywords:** GRP94, brain metastasis, endoplasmic reticulum stress, fatty acids

## Abstract

Metabolic adaptation may happen in response to the pressure exerted by the microenvironment and is a key step in survival of metastatic cells. Brain metastasis occurs as a consequence of the systemic dissemination of tumor cells, a fact that correlates with poor prognosis and high morbidity due to the difficulty in identifying biomarkers that allow a more targeted therapy. Previously, we performed transcriptomic analysis of human breast cancer patient samples and evaluated the differential expression of genes in brain metastasis (BrM) compared to lung, bone and liver metastasis. Our network approach identified upregulation of glucose-regulated protein 94 (GRP94) as well as proteins related to synthesis of fatty acids (FA) in BrM. Here we report that BrM cells show an increase in FA content and decreased saturation with regard to parental cells measured by Raman spectroscopy that differentiate BrM from other metastases. Moreover, BrM cells exerted a high ability to oxidize FA and compensate hypoglycemic stress due to an overexpression of proteins involved in FA synthesis and degradation (SREBP-1, LXRα, ACOT7). GRP94 ablation restored glucose dependence, down-regulated ACOT7 and SREBP-1 and decreased tumorigenicity in vivo. In conclusion, GRP94 is required for the metabolic stress survival of BrM cells, and it might act as a modulator of lipid metabolism to favor BrM progression.

## 1. Introduction

Glycoprotein 96 (gp96, GRP94, Erp99, endoplasmin) is an endoplasmic reticulum (ER) lumen resident protein from the HSP90 family [1,2] that belongs to a set of ER chaperones originally discovered as proteins induced by glucose deprivation [3]. GRP94 often functions as a dimer providing a platform for the assembly, folding or oligomerization of loaded protein cargo [4], and its function is ATP-dependent [5]. GRP94 can be modulated by interferons [6] and by a multitude of stress situations that perturb ER function. One of its most relevant functions is stress resistance, promoting survival and protecting cells against ER stress-induced death [7] in conditions of glucose starvation and/or oxidative stress. GRP94-mediated endoplasmic reticulum stress-resistance phenotype (ERSRP) integrates a set of adaptation pathways that regulates transcription and translation of genes in an attempt to restore functions and re-establish homeostasis [8].

Chaperone overexpression is a general phenomenon that happens in tumor cells as a consequence of the increased proliferation rate and the harmful environment [9]. GRP94 has been associated with cellular transformation in a variety of cancer cell lines, rodent tumor models and human cancer biopsies correlating with increased tumorigenicity [10]. In fact, GRP94 is upregulated in CD44^hi^/CD24^lo^ breast cancer stem cells, which are usually considered tumor-initiating cells with enhanced invasive properties and radiation resistance [11]. Thus, GRP94 might respond to environmental stress mediating the induction of specific ER-proteins, such as heat shock proteins and other glucose-regulated proteins, and allowing tumor-microenvironment cross-talk and metastatic growth [12,13]. Moreover, GRP94 is coupled with secretory pathways and transport of membrane proteins to the cell surface, such as integrins and toll-like receptors, contributing to regulation of immunity and metastasis progression [14,15].

The ER is also the main site for sterol and phospholipid synthesis, since many enzymes of the pathway reside in this organelle [16]. The ER stress response and specifically the unfolded protein response (UPR) pathways are closely associated with lipid metabolism through inositol-requiring enzyme 1α (IRE1α)/XBP1 or activating transcription factor 6, which can enhance phospholipid biosynthesis under ER stress conditions [17]. Therefore, ER stress has been considered an important regulator of lipid metabolism in organs like the liver [13], modulating the expression of key enzymes in the pathway [16]. Furthermore, cancer cells require a constant supply of lipids for membrane synthesis and energy production [18], suggesting ER stress and lipid metabolism might be two processes tightly connected to promote tumor growth. For example, stress derived of poor perfusion and reduced access to exogenous nutrients within the tumor microenvironment can modulate de novo fatty acid synthesis enzymes in cancer cells. Recently, the ER chaperone GRP78 has been described to regulate lipid metabolic enzymes in pancreatic cancer, leading to lipid accumulation after GRP78 depletion [19]. This link between ER stress and metabolic rewiring might be even more important in the pathogenesis of brain metastasis (BrM) due to the different energetic substrates and nutrients in the central nervous system (CNS) environment.

Brain metastasis occurs as a consequence of the systemic dissemination of tumor cells, a fact that correlates with poor prognosis and high morbidity in lung (36–64%), breast (15–25%), and melanoma (5–20%) [20]. We described that GRP94 overexpression might be a hinge orchestrating ERSRP responses in BrM through activation of PERK, ATF6 and IRE1 leading to stress resistance in the hypoglycemic environment of the CNS [21,22]. Here, we investigated the role of GRP94 as a promotor of cell survival and adaptive mechanisms, ameliorating ER stress of brain metastatic cells.

## 2. Results

### 2.1. Brain Metastatic Cells Increase Total Fatty Acid (TFA) and Decrease Total Unsaturated Fatty Acid (TUFA) Content

We previously defined the ERSRP of BrM cells based on a set of brain metastasis biopsies, which are freely available from the Gene Expression Omnibus (GEO) repository (GSE11078) [23]. This organ-specific metastasis signature contained 1193 genes, which clearly distinguished brain from other different metastases [24]. Protein folding enzymes and chaperones appeared as a hub connecting different functions that mediated ERSRP, GRP94 being one of the central proteins [22]. Among the organ-specific deregulated functions in brain metastases, we found three upregulated proteins (Table 1) with a role in fatty-acid (FA) synthesis (PCYT2, SLC25A1) and hydrolysis (ACOT7). These results suggested that BrM presented an organ-specific enrichment in genes modulating FA metabolism. Previous work from our lab identified a role for GRP94 and the ERSRP in mediating brain metastasis [22]. In fact, GRP94 was overexpressed in a brain metastatic variant from breast cancer (435-Br1) compared to the parental cell line (435-P), while there were no differences in GRP78 expression, another chaperone involved in the UPR response (Supplementary Figure S1) and previously linked to lipid metabolism [19,25].

**Figure 1:**
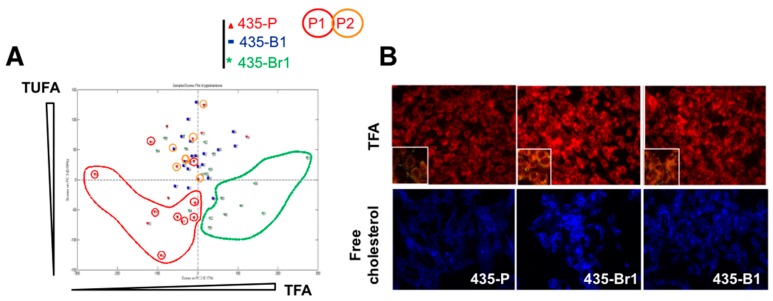
Brain metastatic cells present an organ-specific increase in fatty acid and cholesterol content. (**A**) Raman spectroscopy and PCA analysis of total fatty acids (TFA, 2845 cm^−1^) and total unsaturated fatty acids (TUFA, 3015 cm^−1^) of 435-P, 435-Br1 and 435-B1 cells. (**B**) Lipid content was analyzed by fluorescent staining with Nile red (TFA) and filipin (cholesterol). Nile red’s emission shifts to red when it associates to phospholipids (40×). Insert shows lipid droplets.

Given the role of ER stress in lipid metabolism, we hypothesized that FA metabolism might be differentially enriched in 435-Br1 cells as a consequence of their ERSRP and GRP94 overexpression. Therefore, we quantified intracellular lipid content by Raman spectroscopy (RS) and principal component analysis (PCA). RS is a technique based on the inelastic dispersion of monochromatic radiation which provides specific vibrational signatures of chemical bonds, suitable for the analysis of saturated and unsaturated fatty acids [26]. To verify if the lipid phenotype of BrM was an organ-specific trait, we carried out the analysis of total fatty acids (TFA) and total unsaturated fatty acid (TUFA) in 435-P and 435-Br1 cells, as well as in a metastatic variant to bone (435-B1), and we established a TFA/unsaturated ratio (TFA/TUFA) according to the spectroscopic quantification of TFA (2845 cm^−1^) and TUFA bands (3015 cm^−1^). Analysis of 435-P cells (Figure 1A) revealed a high dispersion in TUFA content and the presence of two unexpected subpopulations (P1 and P2), clearly differentiated by the lower TUFA/TFA ratio of P1 with regard to P2 (*p* < 0.00001). P1 TFA appeared in the same region as 435-Br1 cells while P2 TFA matched 435-B1 cells, suggesting that TUFA/TFA ratio could differentiate metastatic organ specificity. We concluded that 435-Br1 was the cell line with higher TFA content and lower percentage of TUFA, therefore with predominant saturated FA profile while 435-B1 were enriched in TUFA.

To confirm these results, we visualized the amount of TFA by means of the fluorescent stain Nile red [27] (Figure 1B, upper panel). As expected, 435-Br1 cells presented a higher Nile red staining intensity with regard to 435-P and 435-B1 cells. Since increased UPR activation and presence of TFA have been related to cholesterol retention in the ER [28], as well as malignancy, migration, invasion and angiogenesis [29], we analyzed free cholesterol distribution with filipin, a fluorescent probe that binds selectively to free cholesterol but not esterified cholesterol [30]. The high filipin staining intensity in 435-Br1 cells confirmed an increase in free cholesterol content compared to 435-P and 435-B1 cells (Figure 1B, bottom panels).

**Figure 2:**
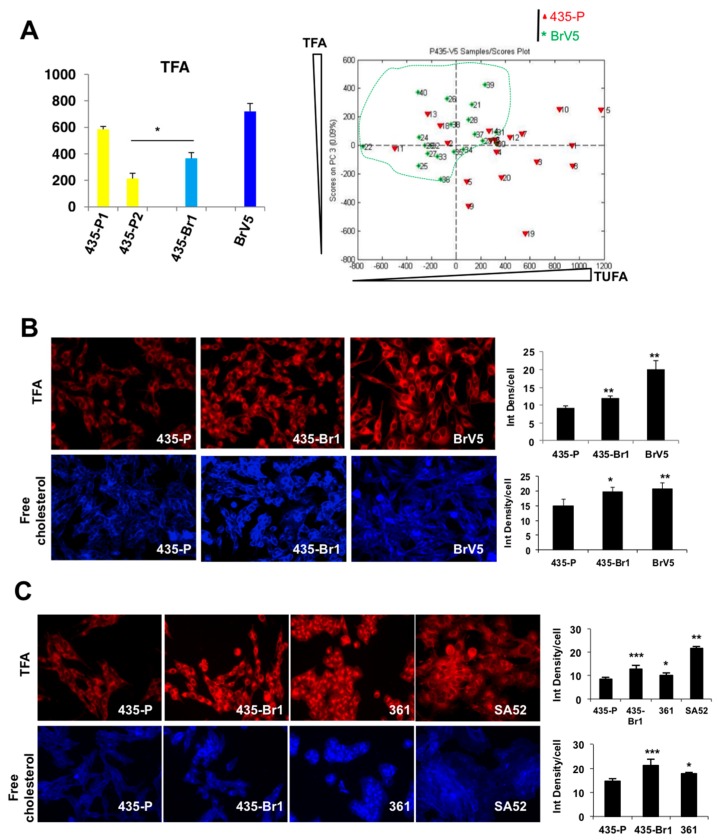
The lipid phenotype is conserved in other brain metastatic cell lines. 435-Br1 cells were obtained after five rounds of in vivo/in vitro passages to obtain a highly brain-specific cell line BrV5. (**A**) TFA were analyzed by Raman spectroscopy (left) and principal component analysis showing an enrichment in TFA content and a decrease in TUFA in BrV5 cells. Error bars represent SD of technical replicates (one representative of two experiments). (**B**) Nile red (top) and filipin (bottom) staining in 435-P and brain metastasis (BrM) cells 435-Br1 and BrV5 (40×, one representative of three experiments). Integrated density was quantified with Image J and normalized to cell number (error bars represent SD of technical replicates, >150 cells). (**C**) Nile red (top) and filipin (bottom) staining in 435-P and BrM cells 435-Br1, MDA-MB-361 and SA52. For Nile red, error bars represent SD of *n* = 2 (435-P, 361, SA52) or *n* = 4 (435-Br1) independent experiments. For filipin, pictures at 40×, one representative of two experiments, >150 cells analyzed. Error bars represent SD of technical replicates. Quantification was not possible for SA52 due to unclear nuclear visualization. For all panels, significance determined with *t*-test. * *p* < 0.05, ** *p* < 0.01, *** *p* < 0.001.

Similarly, RS and PCA analysis were performed in a more enriched organ-specific metastatic cell line to brain (BrV5) obtained after five consecutive in vitro/in vivo selection rounds from 435-Br1 cells, to understand if high lipid content and saturation were determinants of brain metastasis. BrV5 cells presented the highest TFA levels compared to 435-Br1 (366.1 ± 44.1) and 435-P1/P2 cells (Figure 2A, left). Furthermore, PCA analysis differentiated 435-P cells from BrV5 by TFA and TUFA content, with BrV5 presenting the highest amount of TFA and lower TUFA, both properties related to aggressiveness [31]. Thus we reproduced the low levels of TUFA in 435-Br1 and BrV5 cells (*p* = 0.0003) and the differences in TUFA between 435-P1, 64.2 ± 3.6 and 435-P2 cells, 45 ± 1.27, *p* = 0.0023.

Next, we validated these results by Nile red and filipin staining (Figure 2B), confirming that 435-Br1 and BrV5 had a higher Nile red staining compared to 435-P (*p* = 0.003, and *p* = 0.002, respectively). Furthermore, free cholesterol levels were also higher in 435-Br1 and BrV5 compared to 435-P cells (*p* = 0.019 and *p* = 0.005, respectively). To discard any possible deviation of the cellular model, we performed TFA and cholesterol staining in two more brain metastatic variants, MDA-MB-361 and SA52 (Figure 2C). TFA were generally upregulated in all cell lines compared to 435-P (MDA-MB-361 vs 435-P, *p* = 0.037; SA52 vs. 435-P *p* = 0.004). Free cholesterol was also increased in MDA-MB-361 compared to 435-P cells (*p* = 0.01).

**Figure 3:**
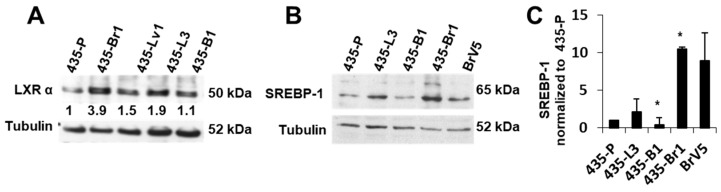
BrM cells upregulate genes involved in FA synthesis. (**A**,**B**) Immunoblotting of LXRα (**A**) and SREBP-1 (**B**) in metastatic variants. (**C**) SREBP-1 expression was quantified vs. tubulin and normalized to 435-P cells. Error bars represent SEM of three independent experiments. Significance determined with *t*-test. * *p* < 0.05.

Finally, to assess if the increase in FA levels was related to increased synthesis, we analyzed the expression of two genes: Sterol-Regulatory Element-Binding Protein 1 (SREBP-1), a nuclear factor upstream of ACC or FASN [32,33] and Liver X Receptor (LXRα), an oxysterol-induced nuclear factor, which regulates SREBP-1 expression driving lipid metabolism downstream of insulin/AKT signaling in the liver [34,35]. LXRα was overexpressed in 435-Br1 compared to 435-P and other metastases (Figure 3A). SREBP-1 expression was in accordance to LXRα levels, as brain metastatic cells (435-Br1 and BrV5) showed an increase in this protein compared to other metastases (Figure 3B). Similar to what we observed with FA levels, bone metastasis behaved like 435-P cells, with the lowest LXRα and SREBP-1 expression. Lung metastatic cells showed an intermediate phenotype between 435-P and 435-Br1 cells. All together these data suggested that brain metastatic cells displayed a lipogenic phenotype differential among metastatic cells to other organs.

### 2.2. Inhibition of FA Import to the Mitochondria Impairs BrM Cell Survival after Metabolic Stress

**Figure 4:**
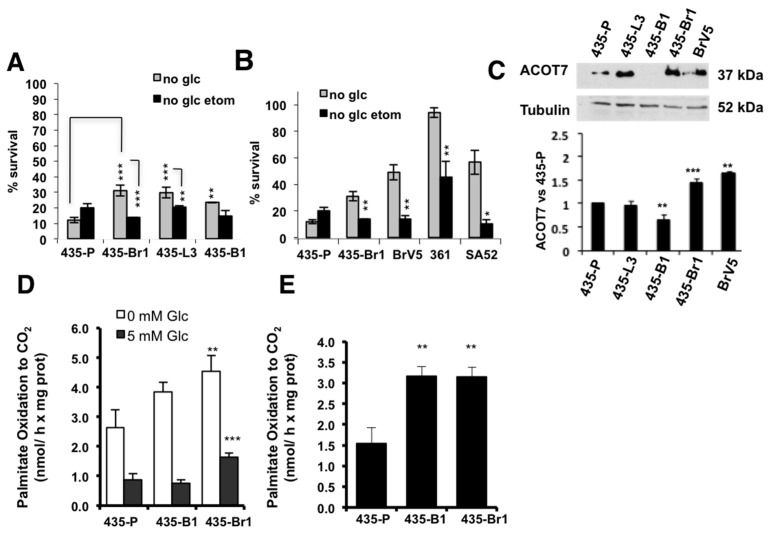
BrM cells are more sensitive to etomoxir in glucose deprivation conditions. (**A**,**B**) Survival of 435-P and a set of metastatic cells to different organs (**A**) or to brain (**B**) cultured without glucose ± etomoxir (200 µM). Bars represent relative survival of each cell line vs. its control (non-treated) with glucose, error bars represent SD of independent experiments (*n* = 3). (**C**) ACOT-7 expression was quantified vs. tubulin (error bars represent SEM of *n* = 4 (435-P, 435-Br1, 435-B1), *n* = 5 (435-L3) or *n* = 3 (BrV5) independent experiments) (**D**) Palmitate oxidation was calculated after adding ^14^C-palmitate and measuring released ^14^CO_2_ production at 5 or 0 mM glucose (error bars represent SEM of three independent experiments). (**E**) Palmitate oxidation ratio at 0 mM glucose vs. 5 mM glucose (error bars represent SEM of 3 independent experiments). For all panels, significance determined with *t*-test. * *p* < 0.05, ** *p* < 0.01, *** *p* < 0.001.

Metastatic cells may undergo a variety of bioenergetic alterations including shift to more glycolytic or more oxidative metabolism. Our results suggested that brain metastatic cells accumulate FAs; therefore, we hypothesized that FAs, essential for the biosynthesis of membranes, might also be required as bioenergetic substrates in BrM cells. To analyze if FA could be used as an alternative energy source in conditions of glucose restriction, we deprived cells of glucose and pyruvate for 72 h maintaining glutamine (2 mM) and serum (10%) concentration (Figure 4A). Glucose deprivation impaired survival (15%) of 435-P cells differentially with regard to metastatic cells in brain 435-Br1 (30%), lungs 435-L3 (30%) and bone 435-B1 (25%). To assess the importance of FA oxidation (FAO) in survival of BrM cells, we used etomoxir to inhibit the mitochondrial carnitine palmitoyltransferase I (CPT1), a transporter that mediates FA import to the mitochondria and is the rate-limiting step of FAO [36]. Etomoxir treatment in glucose starved cultures decreased survival of 435-Br1 (*p* = 0.001) and to a lesser extent in 435-L3 (*p* = 0.031) with regard to 435-P cells (Figure 4A). The same experiment performed in the set of brain metastatic cells (BrV5, MDA-MB-361, SA52) proved the increased resistance to glucose deprivation in all these cell lines compared to the parental cells, as well as impaired survival in glucose deprivation after etomoxir (Figure 4B). These results suggested that brain metastatic cells might use FA as an alternative energy source in glucose restriction conditions, whereas other metastatic cells may depend on FA import to a lesser extent.

Our transcriptional studies showed an increased expression of ACOT7 in brain metastatic cells, an enzyme that hydrolyzes palmitoyl-CoA under the control of a sterol response element (SRE) [37]. Since palmitoyl-CoA is the substrate of CPT-I, it is plausible to hypothesize that ACOT7 could modulate lipid import to the mitochondria and FAO. ACOT7 was differentially overexpressed in 435-Br1 and BrV5 cells compared to bone metastasis or 435-P (Figure 4C), suggesting a possible induction of mitochondrial FAO in brain metastasis and reinforcing the similarities between the parental and bone metastatic subtype. To further evaluate the importance of the mitochondria in the metabolic features observed so far, we assessed mitochondrial morphology in the set of metastatic variants. Mitochondrial dynamics is characterized by fusion and fission events that can regulate mitochondrial function [38,39]. Therefore, we performed fluorescent staining with Mitotracker Green (Supplementary Figure S2A) to evaluate mitochondrial morphology. 435-P cells showed low staining intensity and fragmented mitochondria in the perinuclear region while the BrM variants presented higher staining and elongated mitochondria (Supplementary Figure S2A). The rest of metastatic variants to lung and bone presented an intermediate phenotype. To quantify the differential phenotype between 435-P and BrV5, we calculated mitochondrial elongation (see Methods) [40] and we classified cells in three subtypes according to elongation levels: fragmented, intermediate and tubular (Supplementary Figure S2B). BrV5 cells had a higher content of tubular mitochondria compared to 435-P (48% vs. 28%), while 435-P cells presented mainly fragmented and intermediate mitochondria. Mitochondrial fusion correlates with increased oxidative phosphorylation and mitofusin expression (Mfn1, Mfn2) [41]. BrM cell lines overexpressed Mfn2 (Supplementary Figure S2C), a protein that has been described to promote ER-mitochondria contact modulating Ca^2+^ homeostasis, glucose oxidation and Krebs cycle [41]. Mfn1 and Mfn2 were down regulated in 435-P correlating with a fragmented phenotype, together with decreased VDAC expression (Supplementary Figure S2C), which suggested a lower mitochondrial content and function in this cell line.

Finally, we analyzed FAO (fatty acid oxidation) after glucose withdrawal by means of a radioactive assay that consists in addition of ^14^C-palmitate (Figure 4D) and measurement of CO_2_ production. In the presence of glucose (5 mM), 435-Br1 cells showed an increased release of CO_2_ compared to 435-P (Figure 4D, left). In glucose deprivation conditions, all cell lines induced ß-oxidation, but these differences were more significant in 435-Br metastatic cells compared to 435-P (Figure 4D). Moreover, we calculated the net increase in oxidation subtracting the values of released CO_2_ at 0 mM glucose vs. 5 mM (Figure 4E), which proved that induction of FA oxidation in response to glucose deprivation differentially occurs in metastatic cells vs. 435-P. All together these results suggested that metastatic cell lines might respond to metabolic stress inducing fatty acid oxidation, even though other factors like ability to face ER stress or FA availability for membrane synthesis might limit survival.

### 2.3. GRP94 Is Required for Tumor Growth In Vivo and It Modulates the Lipogenic Phenotype of Brain Metastatic Cells

**Figure 5:**
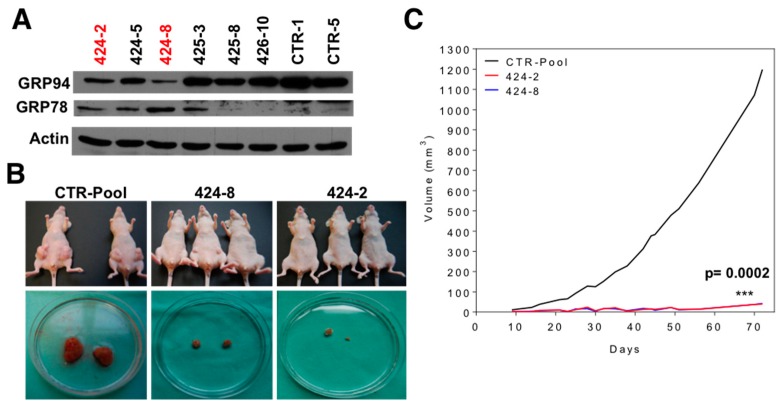
Glucose-regulated protein 94 (GRP94) deletion reduces tumorigenic efficiency in an orthotopic model. (**A**) Immunoblotting of GRP94 and actin shows efficient GRP94 knockdown in BRV5CA1 cells. (**B**) Nude mice were injected in the mammary fat pad with control (BRV5CA1 CTRL-pool) or shGRP94 (424-2 and 424-8) cells. Representative mice are shown presenting a drastic decrease in tumor burden. (**C**) Tumor volume was measured at the indicated times based on caliper measurements of tumor length and width as volume (mm^3^) = (Length^2^ × Width^2^/2). Tumor volume was normalized to the volume of the first observation (day 14) for each group. Significance was determined with *t*-test. *** *p* = 0.0002.

Since the UPR and ER stress have been previously related to lipid metabolism [13,16,19,28,33,42], we hypothesized that overexpression of GRP94 might be associated with the increased fatty acid content and FAO observed in brain metastatic cells. To evaluate the role of GRP94 in FA synthesis and degradation, we generated stable knock-downs of GRP94 in the brain metastatic BRV5CA1-GFP cell line (see Material and Methods). Interestingly, GRP94 ablation induced a compensatory activation of GRP78, as previously described [43] (Figure 5A). We pooled several control clones (CTR-pool) and selected two GRP94 clones shGRP94-2 (424-2) and shGRP94-8 (424-8) for orthotopic injection. The growth of shGRP94 cells was similar to parental and BRV5CA1 cells at 48, 72 and 96 h in standard culture conditions (data not shown). 424-2 and 424-8 cells showed a decreased tumorigenicity and growth in vivo with regards to CTRL-pool (Figure 5B). Furthermore, tumor volume was dramatically decreased after GRP94 ablation compared to the control, *p* = 0.0002 (Figure 5C) demonstrating that GRP94 is required for in vivo tumor growth.

**Figure 6:**
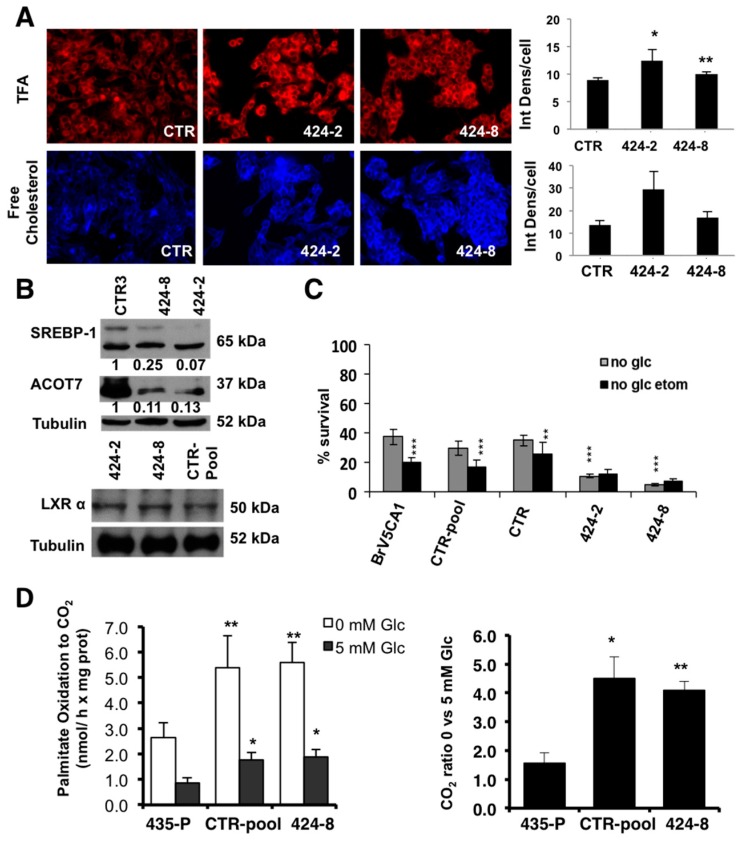
GRP94 ablation impairs survival in glucose restriction conditions and increases lipid content. (**A**) Lipid content was analyzed by fluorescent staining with Nile red (TFA) and filipin (cholesterol) for V5CA1-CTR, 424-2 and 424-8 cells (40x, one representative experiment of three, error bars represent SD of >180 cells analyzed). (**B**) SREBP-1 and LXRα expression was quantified vs. tubulin. (**C**) Survival of BrM metastatic cells after GRP94 ablation cultured without glucose ± etomoxir (200 µM). Bars represent relative survival of each cell line vs. its control (non-treated) with glucose, error bars represent SD of three independent experiments. (**D**) Palmitate oxidation was calculated after adding ^14^C-palmitate and measuring released ^14^CO_2_ production at 5 or 0 mM glucose (error bars represent SEM of three independent experiments). (right) Palmitate oxidation ratio at 0 mM glucose vs. 5 mM glucose. For all panels, significance determined with *t*-test. * *p* < 0.05, ** *p* < 0.01, *** *p* < 0.001.

To analyze if the metabolic changes observed in GRP94 overexpressing cells might be reversed in GRP94 ablated cells, we measured TFA and cholesterol content by fluorescent staining (Figure 6A). Nile red revealed increased phospholipid content in shGRP94 V5CA1 cells compared to the control (clone 2, *p* = 0.015, clone 8, *p* = 0.007), together with a trend to increased cholesterol content. Next, we analyzed protein changes secondary to down-regulated GRP94 (Figure 6B). GRP94 depletion induced a significant down-regulation of ACOT7 and SREBP-1 in ablated cells with no differences in LXRα expression between depleted GRP94 clones and controls, suggesting lipid accumulation might be independent of these specific synthesis pathways. Moreover, we analyzed if GRP94 ablation decreased cell ability to survive in hypoglycemia (Figure 6C). Interestingly, shGRP94-2 (424-2) and shGRP94-8 (424-8) cells were more sensitive to glucose deprivation with a lower survival ratio than control cells (values normalized to each cell line in glucose conditions), reinforcing the role of GRP94 as a mediator of survival in glucose starvation. Etomoxir treatment did not impair survival, suggesting that GRP94 ablated cells were no longer dependent on mitochondrial FA import (Figure 6C). To test this hypothesis, we also measured FAO and we observed no differences in palmitate oxidation secondary to GRP94 ablation (Figure 6D). Although further studies are required to determine the mechanism, these data suggest that GRP94 modulates FA accumulation while it doesn’t impact fatty acid oxidation. Overall, our work identifies a lipid phenotype consisting of increased FA content and saturation common in brain metastatic cells. Furthermore, we identified GRP94 as a key mediator of BrM metabolic stress survival, likely through regulation of the ER stress response, and we describe a previously unknown role of GRP94 in lipid metabolism.

## 3. Discussion

The increase in the biosynthesis of new molecules as well as the capacity to fulfill the energetic requirements of the cell are crucial for metastatic progression [44], but at the same time these processes generate a permanent state of energy demand and metabolic stress [45]. The activation of the cancer stress phenotype [46] allows the correct adaptation to this stress-activating pathways to promote cell survival in a process defined as “non-oncogene addiction” or non-oncogene co-dependency [47]. Here we show that expression of GRP94 promotes a rewiring of cellular metabolism in BrM cells conferring a survival advantage in glucose deprivation conditions by activating lipid metabolism and glucose-independent growth. These might be crucial to brain metastasis progression given the unique metabolic features of the brain interstitial space, which include lower glucose levels [48] and cholesterol enrichment by the novo synthesis that compensates for the inability of cholesterol to cross the blood–brain barrier [49]. Our results also suggest that BrM cells increase cholesterol metabolism, which may act as an endogenous ligand for LXRs [50], mimicking the tissue-specific homeostasis.

Moreover, the use of RS and PCA allowed an accurate analysis of cellular lipid content, differentiating cells by their metabolic profile [51]. In particular, we describe a lipogenic phenotype (increased TFA/TUFA ratio) associated to brain metastasis vs. bone metastasis. This increase in fatty acid content likely correlates with FA synthesis given the overexpression of proteins like SREBP-1 and LXRα, which induce ACC and ACLY transcription [34,35] and activate other pro-synthetic genes like FASN [52]. Increased fatty acid synthesis is a bad prognostic factor in breast cancer [53,54] as it confers resistance to therapy [55,56] and promotes metastatic and invasive capacity [51,57]. Our results are in accordance to previous studies that showed a correlation between ACOX1 and FASN expression and organ specificity to brain in a set of patients’ metastasis [58]. RS may be considered a promising strategy to stratify organ-specific metastasis according to lipid content and may be used to select patients that could benefit from therapies targeting lipid metabolism.

GRP94 deletion induced further FA accumulation but it didn´t impair fatty acid oxidation. One possible explanation is that GRP94 ablation led to activation of FA import pathways that collaborate in increasing intracellular FA content. On the other hand, we cannot exclude the possibility that the lipid phenotype of BrM cells was not exclusively dependent of GRP94 levels. In fact, it may involve the action of other stress chaperones like GRP78, which was overexpressed in GRP94 ablated cells, and other UPR proteins that might in turn promote FA synthesis [7]. GRP78 has been associated with lipid accumulation in breast cancer cells due to an inhibition of mitochondrial fatty acid transport, resulting in a reduction of FAO [25]. Since we didn’t observe defects in FA oxidation after GRP94 deletion, this is unlikely to be the main mechanism leading to FA accumulation. In addition, GRP78′s role as a lipid regulator is not only restricted to FAO as it has been described to regulate SREBP-1 function among others [59]. Since both chaperones have been previously reported as co-regulated under a variety of stress conditions, further studies are needed to understand the role of these chaperones in lipids metabolism and metastasis progression.

Interestingly, despite FA accumulation after GRP94 KD, we observed a decrease in SREBP-1 levels, the master regulator of fatty acid and triacylglycerol synthesis and a mediator of BRAF-targeted therapy resistance [60]. Since metabolism includes multiple pathways that are tuned to meet the increasing metabolic requirements during the metastatic process [61], it is possible that FA synthesis could be decoupled from SREBP-1 and engage downstream effectors like ACC-1 or FASN. GRP94 expression is critical in chaperoning lipogenesis regulator genes. Since SREBP-1 is transported from the ER to the Golgi, where it is consecutively cleaved by two proteases to be transcriptionally active [62], further experiments would be required to understand if SREBP-1 may be a client protein affected by the chaperone function of GRP94.

One of the most dramatic differences induced by GRP94 depletion in BrM cells resides in the down-regulation of ACOT7, a major isoform of the ACOT family that catalyzes hydrolysis of fatty acyl-CoAs to free fatty acids that may condition the recruitment of FA to be metabolized in the mitochondria. High expression of acyl-CoA thioesterases has been implicated in regulatory mechanisms involved in ER stress in cancer [63]. Moreover, low levels of ACOT7 prevented human breast cancer by activating p53-p21 signaling pathway and cell cycle-arrest [64]. Further studies are required to dissect the relationship between the observed down-regulation of ACOT7 in GRP94 ablated cells and low tumorigenicity.

Different tumoral subtypes acquire different bioenergetic alterations, either towards a more glycolytic or more oxidative metabolism. This tissue-specific context invalidates the idea of a unique common transition in all cancer types, as Warburg hypothesized [65]. Our results confirmed that brain metastatic cells were more resistant to glucose deprivation than the parental cell line and other metastasis. These data, in addition to etomoxir susceptibility and mitochondrial morphology, suggested that carcinoma cells growing in the brain might be more dependent on mitochondrial metabolism, mainly through FA import and oxidation. This metabolic shift is in agreement with previous studies showing gain of glucose-independent growth [48] and increased oxidative phosphorylation and beta-oxidation in breast cancer BrM [66]. The arrival and establishment in the new microenvironment might give preference to some metabolic functions in an organ-specific manner, and maintenance of oxidative metabolism may be critical for BrM establishment and progression. Further work is required to evaluate the role of GRP94 in maintaining respiration and ATP production [67] in BrM. From these studies, we conclude that GRP94 is critical in the metabolic reprogramming of BrM cells, rewiring lipid metabolism and mediating glucose-independent survival during metastasis progression. Further work using untargeted lipidomic analysis of brain metastatic cells might reveal lipid species altered favoring metastasis progression.

## 4. Materials and Methods

### 4.1. Cell Culture and Treatments

MDA-MB-435 (435-P) breast cancer cells and their metastatic variants to brain (435-Br1), lung (435-L3), bone (435-B1) and liver (435-Lv1) were obtained from successive in vivo/in vitro passages [68]. MDA-MB-361 cells were obtained from Laboratoire d’Oncogénetique (Centre Rue Huguenin, Saint-Cloud, France). SA52 cells were obtained from Laboratory of Experimental Cancerology (University of Ghent, Ghent, Belgium). BrV5 and BRV5CA1 were obtained after several rounds of in vitro/in vivo injection of BR-eGFP-CMV/Luc cells, as described elsewhere [69]. All cell lines were maintained under standard conditions in DMEM/F12 medium (Invitrogen, Carlsbad, CA, USA) supplemented with 10% FBS, 1 mM pyruvate and 2 mM L-glutamine at 37 °C in a humidified 5% CO_2_ incubator. For glucose deprivation experiments, cells were seeded and after 24 h, they were starved of glucose (0 or 4.5 mg/mL) using DMEM without glucose (Invitrogen) supplemented with 10% SBF and L-Gln 2 mM for 48 or 72 h. Treatment with etomoxir (Sigma, St. Louis, MO, USA) was performed at a final concentration of 200 μM for 72 h.

### 4.2. Cell Viability

Cells were fixed in paraformaldehyde 4% and stained with crystal violet (0.2% in 2% ethanol) for 20 min. Dye was extracted with SDS 10% and relative proliferation was determined by measuring OD at 595 nm to evaluate survival.

### 4.3. Fluorescent Stainings

Nile red and filipin staining: 6–8 × 10^4^ cells were seeded in 24-well plates and left to adhere for 24 h on coverslips coated with gelatin 2%. Cells were fixed in paraformaldehyde 4%, washed with PBS1X and stained with Nile red (Sigma, 1 μg/mL in PBS 1X) or filipin (Sigma, 20 μg/mL in PBS 1X) for 1 or 2 h, respectively. Coverslips were mounted with Vectashield with (Nile red) or without (filipin) DAPI (Vector laboratories). Samples were visualized with an Olympus BX60 (Olympus, Tokyo, Japan) microscope at 40× or 60×. Integrated density was quantified with Image J using a constant area and was normalized to cell number (Int Dens/cell). Immunofluorescence: For immunofluorescence, 1 × 10^5^ cells were seeded in 24-well plates containing gelatin-coated cover slips and they were fixed after 24 h using paraformaldehyde 4% for 15 min at 4 °C. Blocking and permeabilization was performed using PBS1x-20%FBS containing 0.2% Triton X-100 for 30 min at room temperature. The antibodies used were: GRP94 (1:800, Santa Cruz, CA, USA), and GRP78 (1:400, Santa Cruz). Alexa Fluor^®^ 555 anti-mouse and Alexa Fluor^®^ 488 anti-goat (Invitrogen) were used as secondary antibodies at the adequate concentration (1:1000). Vectashield (Vector laboratories, Burlingame, CA, USA) with DAPI was used for nucleus visualization. Mitochondrial staining with Mitotracker green: Cells were stained with Mitotracker green (M-7514, Molecular probes Invitrogen) at 100 nM for 30 min, washed and analyzed in complete media without fixation in a confocal microscope (SP2 Leica, 63×, excitation at 488 nm, emission at 500–540 nm). Elongation was calculated with Image J (v 1.52a) as previously described. Briefly, images were transformed to greyscale and inverted, threshold was set to 90 and the function Analyze Particles was used to obtain circularity values (values of 1 were excluded). Elongation was defined as the inverse of circularity. Frequency analysis was used to define the abundance of each phenotype normalized to total number of mitochondria analyzed. An average of 90 cells were analyzed for each cell line.

### 4.4. Stable GRP94 Protein Knockdown

GRP94 stable knock-down: Five constructs containing different short hairpin RNA (shRNA) against GRP94 (TRCN0000029424, TRCN0000029425, TRCN0000029426, TRCN0000029427, TRCN0000029428) and a non-target shRNA control vector, were obtained commercially from Sigma Aldrich MISSION^®^ shRNA. BRV5CA1-GFP cells were transfected with the five shGRP94 constructs or shControl, respectively, using Lipofectamine 2000 (Invitrogen) as a transfection agent. Clonal selection was performed and Western blot and immunofluorescence were used to validate GRP94 knockdown.

### 4.5. Protein Expression

To assess protein expression in the different experiments, cell lines were lysed in a 1% SDS (v/v) extraction buffer containing an anti-protease cocktail (Roche, Vilvoorde, Belgium). Protein concentrations were determined using the Bradford assay (MicroBCA, Pierce, Belgium). After resolution by SDS-PAGE, electrophoresed proteins were transferred to polyvinylidene fluoride (PVDF) membranes that were blocked and probed with the following antibodies: ACOT7 (1:1000, Abcam, Cambridge, UK), LXRα (1:1000, PPMX, Tokyo, Japan), SREBP-1 (1/500, Abcam), GRP94 (1/1000, Sta Cruz, CA, USA), Mfn1 (1:1000, Santa Cruz, CA, USA,), Mfn2 (1:2000, Abcam), VDAC (1:5000, Abcam), α-tubulin (1:10,000, Sigma), actin (1:2000, Sigma) and the corresponding peroxidase-conjugated secondary antibody at 1:2000: Peroxidase conjugated anti-rabbit secondary Ab (Amersham, Little Chalfont, UK), Peroxidase conjugated Antimouse secondary Ab (Pierce, Perbio Science Ltd., Cheshire, UK). Immunoreactive bands were quantified using a VersaDoc™ (BioRad Laboratories, Hercules, CA, USA) Imaging System using the Super Signal west-Pico (Pierce Biotechnology, Rockford, IL, USA). MWs were established with See Blue Plus2 prestained Standford (Invitrogen).

### 4.6. ^14^C-Pamitate Oxidation

Fatty acid oxidation was assessed by oxidation of ^14^C-palmitate, as previously described [70]. The 1 × 10^5^ cells were seeded in 12-well plates and left to adhere for 24 h. Cells were washed with 1 mL of 0.1% BSA fatty acid free in KRBH (NaCl 135 mM, KCl 3.6 mM, NaH_2_PO_4_ 0.5 mM, MgSO_4_ 0.5 mM, CaCl_2_ 1.5 mM, NaHCO_3_ 2 mM and HEPES 10 mM, pH 7.4). Cells were incubated in a sealed system with 500 µL of 0.1% BSA fatty acid free in KRBH for 30 min and washed again with the first solution. The mix reaction (0.9 mM carnitine, 0.25 mM palmitate and 1 μCi/mL (1-^14^C) palmitate bound to 1% BSA, in KRBH with (5 mM) or without glucose) was added. Released ^14^CO_2_ was fixed in a Whatman filter moistened in KOH. After 4.5 h at 37 °C in a CO_2_-free incubator, 100 µL of perchloric acid at 40% was injected in the well and left overnight. The potassium hydroxide-saturated filters were immersed in 5 mL of scintillation fluid. Protein quantification for each cell line was performed by BCA assay. Results are expressed as nmol palmitate × mg^−1^ prot × h^−1^ = (cpm sample-cpm blank) × 125/(total cpm × mg prot × h).

### 4.7. Animal Models

Athymic Nude-Foxn1nu female mice weighing 22–28 g were purchased from Harlan Laboratories S.A. (Barcelona, Spain) and were housed in the IDIBELL facility in SFP conditions, at 20–24 °C, 60% relative humidity, and 12 h light and dark periods. Animals were allowed free access to UV-irradiated water and an adequate sterile diet. All animal-related procedures were performed in accordance with the National Institute of Health Guidelines for the Care and Use of Laboratory and with the approval of the animal care committee (Reference 9703 (protocol number)). Mammary tumors were induced by inoculation of 1 × 10^6^ of cells in 0.05 mL of medium without serum in the inguinal mammary gland (IMFP). The xenografts were implanted in animals under isoflurane-induced anesthesia. Tumor volume was measured twice a week based on caliper measurements of tumor length and width as volume (mm^3^) = (Length^2^ × Width^2^/2). Animals were euthanized at day 72 after injection when tumors reached ~1000 mm^3^.

### 4.8. Raman Spectroscopy

For measurements in the 2820–3030 cm^−1^ range, 9 × 10^4^ cells were seeded in Petri dishes with #0 coverglass (Mattek, Ashland, MA). Cells were maintained under standard culture conditions. Hypoglycemia (0 mg/mL) was induced after 24 h of cell seeding and maintained for 48 h until they were analyzed. They were fixed for 15 min at 4 °C with paraformaldehyde at 4% in PBS and, after 3 PBS washes, maintained at 4 °C in PBS. Raman spectra were acquired using an InVia Raman microscope (Renishaw, UK) with a backscattered configuration. Raman excitation was performed with a 514 nm optical beam focused through a 60× objective and with 9 mW power [51]. The spectra were background subtracted with a custom-written Labview program and the Gaussian fits for total fatty acids (TFA) and total unsaturated fatty acids (TUFA) bands (2845 cm^−1^ and 3015 cm^−1^ respectively) were performed in Matlab (Mathworks Inc., Natick, MA) allowing the quantification of the two types of fatty acids in the cytoplasm [26]. Normalization under all Raman spectra was performed to correct for the different amplification in the signal. This normalization can be based on the fact that the spectral region used (the CH stretching region) can be considered as the total biomass present in our confocal volume [71].

### 4.9. Statistical Analysis

Principal component analysis (PCA) was performed over the pre-processed Raman spectra in order to evaluate the spectral differences between the cancerous cell lines studied and to develop a model allowing their discrimination and classification [72]. PCA operates in an unsupervised manner (no previous knowledge of the samples under study is provided) and finds an alternative set of coordinates, the principal components (PCs), to reduce the dimensionality and complexity of the data set. All the spectra can then be explained in a much simpler fashion through a small number of PCs that accounts for the maximum variance in the data [51]. PCA was carried out in Raman experiments in the range of 2820 to 3030 cm^−1^ to differentiate cell groups according to fatty acid content and type. Multivariate statistical analysis was performed using the PLS toolbox (Eigenvector Research, Wenatchee, WA) in the Matlab (Mathworks Inc., Natick, MA) programming environment. SPSS (Statistical Package for the Social Sciences) for Windows was used for the statistics of TFA and TUFA quantification. In all the analyses, differences were considered significant when student’s “*t*” was lower than 0.05.

Student’s *t*-test was used to compare groups of in vitro and in vivo experiments. In all the analyses, differences were considered significant when *p* < 0.05. *p*-Values lower than 0.05 were considered significant. Microsoft Excel and GraphPad were used to plot the graphs and perform statistical analysis.

## Figures and Tables

**Table 1:** Genes differentially expressed in brain metastasis of breast cancer patients compared to other metastases.

Gene	Protein	Location	Function
ACOT7	Acyl coenzyme A thioester hydrolase	Cytoplasmic	Acyl-CoA hydrolysis
PCYT2	Ethanolamine-phosphate cytidylyltransferase	Cytoplasmic	Phospholipid biosynthesis
SLC25A1	Tricarboxylate transport protein	Mitochondrial	Citrate/malate exchange, FA synthesis

## Supplementary Materials

Supplementary materials can be found at https://www.mdpi.com/1422-0067/20/16/3883/s1.

## Abbreviations

GRP94	Glucose-Regulated Protein 94
FA	Fatty Acids
ER	Endoplasmic Reticulum
UPR	Unfolded Protein Response
ERSRP	ER stress-resistance phenotype
IRE1	Inositol-requiring enzyme 1
CNS	Central Nervous system
TFA	Total Fatty Acid
TUFA	Total Unsaturated Fatty Acid
RS	Raman Spectroscopy
PCA	Principal Component Analysis
SREBP-1	Sterol Regulatory Element-Binding Protein- 1
LXR-α	Liver X Receptor
BrM	Brain metastasis
FAO	Fatty Acid Oxidation
Mfn	Mitofusin
KD	Knock-down
