# Supplementary material for: GRP94 Is Involved in the Lipid Phenotype of Brain Metastatic Cells
Source: Int J Mol Sci. 2019 Aug 9;20(16):3883. doi: 10.3390/ijms20163883 (PMC6720951; doi:10.3390/ijms20163883)
Supplement: Supplementary file 1 [file ijms-20-03883-s001.pdf]

Santana-Codina et al, SUPPLEMENTARY FIGURES

Supplementary Figure 1

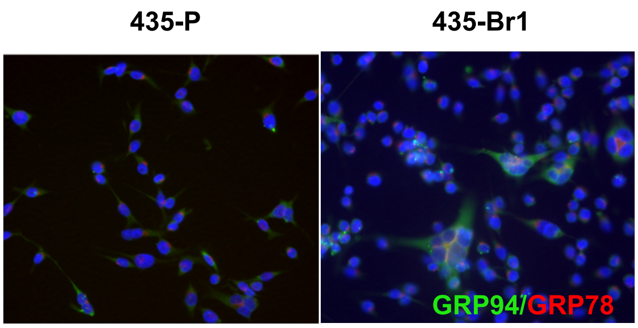

Supplementary Figure 1. Characterization of breast cancer metastatic variants. Immunofluorescence analysis of GRP94 expression (green) and GRP78 (red) in 435-P and 435-Br1 cells. DAPI was used for nucleus visualization.

Supplementary Figure 2

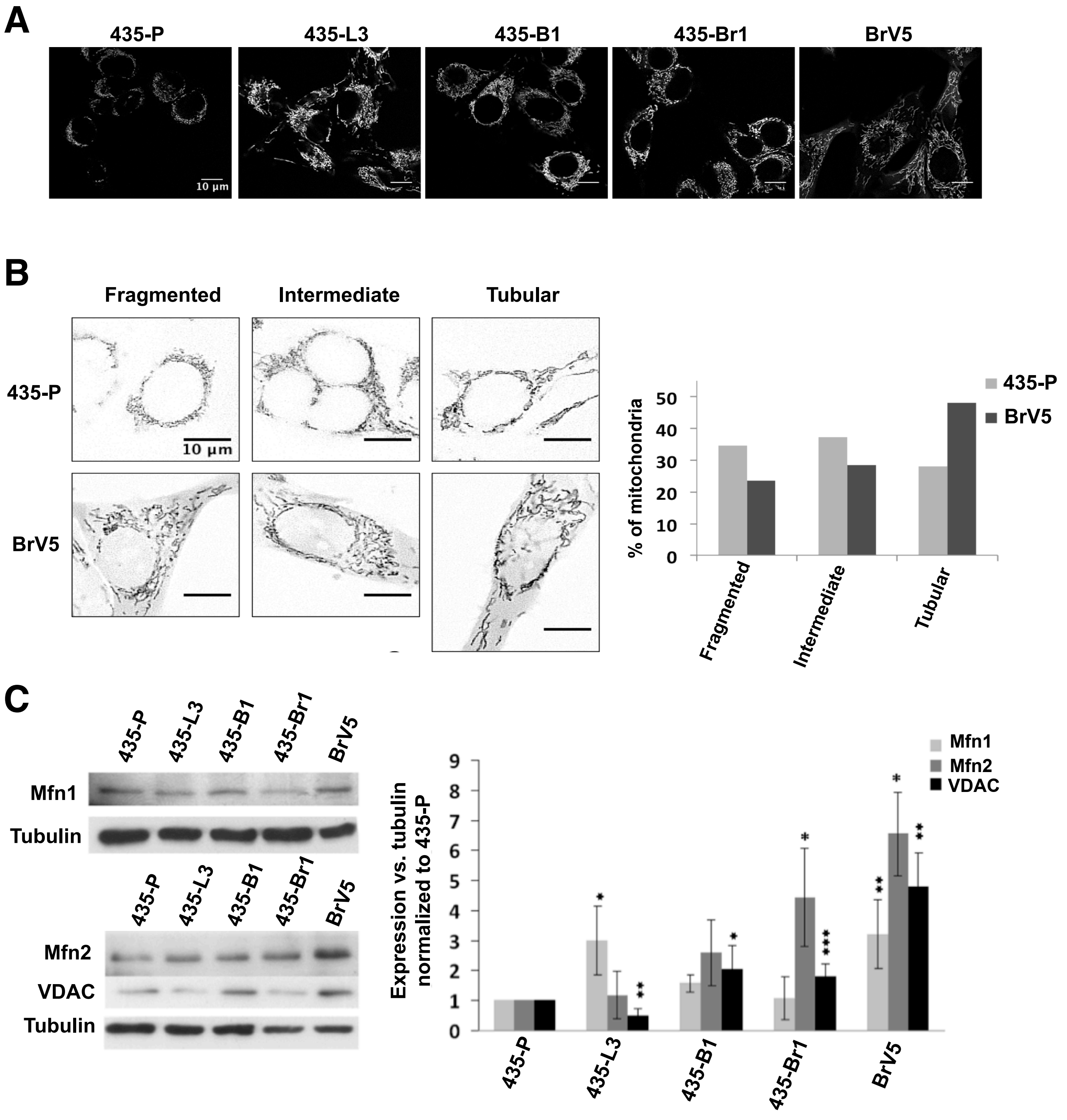

Supplementary Figure 2. Mitochondrial morphology features in metastatic variants. (a) Confocal microscopy of metastatic variants stained with Mitotracker green (63X, 1 representative of 2). **(b)** Elongation was calculated as the inverse of circularity and cells were classified in three subtypes according to mitochondrial elongation: fragmented, intermediate and tubular (85 cells analyzed per group, 1 experiment representative of 2). **(c)** Immunoblotting of Mfn1, Mfn2 and VDAC (left). Expression was quantified to tubulin and normalized to 435-P cells. Error bars represent s.e.m. of 4 independent experiments.
